# Effects of Carboxymethyl Modification on the Acidic Polysaccharides from *Calocybe indica*: Physicochemical Properties, Antioxidant, Antitumor and Anticoagulant Activities

**DOI:** 10.3390/antiox12010105

**Published:** 2022-12-31

**Authors:** Ambika Nataraj, Sudha Govindan, Archana Rajendran, Prasanna Ramani, Krishnamoorthy Akkana Subbaiah, Paulo E. S. Munekata, Mirian Pateiro, José M. Lorenzo

**Affiliations:** 1 Department of Biochemistry, School of Biosciences, Periyar University, Salem 636001, India; 2 Dhanvanthri Laboratory, Department of Sciences, Amrita School of Physical Sciences, Amrita Vishwa Vidyapeetham, Coimbatore 641112, India; 3 Center of Excellence in Advanced Materials & Green Technologies (CoE–AMGT), Amrita School of Engineering, Amrita Vishwa Vidyapeetham, Coimbatore 641112, India; 4 Department of Plant Pathology, Tamil Nadu Agricultural University, Coimbatore 641003, India; 5 Centro Tecnológico de la Carne de Galicia, Avd. Galicia No. 4, Parque Tecnológico de Galicia, 32900 San Cibrao das Viñas, Spain; 6 Área de Tecnoloxía dos Alimentos, Facultade de Ciencias, Universidade de Vigo, 32004 Ourense, Spain

**Keywords:** *Calocybe indica*, polysaccharides, carboxymethylation, antioxidant activity, anticoagulation activity, antitumor activity

## Abstract

An acidic polysaccharide fraction was obtained from *Calocybe indica* (CIP3a) after subjecting it to hot water extraction followed by purification through DEAE-cellulose 52 and Sepaharose 6B column chromatography. The CIP3a was further modified using chloroacetic acid to yield carboxymethylated derivatives (CMCIP3a). The modified polysaccharide was characterized using various spectroscopic methods. In addition, further antioxidant, antitumor and anticoagulant activities were also investigated. The polysaccharides CIP3a and CMCIP3a were heterogeneous in nature and composed of various molar percentages of glucose, arabinose and mannose with molecular weights of 1.456 × 10^3^ and 4.023 × 10^3^ Da, respectively. The NMR and FT-IR data demonstrated that the carboxymethylation on the polysaccharide was successful. In comparison to CIP3a polysaccharides, the modified derivatives had lower sugar and protein contents, and higher levels of uronic acid. The in vitro antioxidant activity showed that CMCIP3a with higher molecular weight displayed an elevated ability in scavenging the DPPH radical, ABTS, superoxide, hydroxyl radical, ferric reducing power, cupric reducing power and erythrocyte hemolysis inhibition with an EC_50_ value of 2.49, 2.66, 4.10, 1.60, 3.48, 1.41 and 2.30 mg/mL, respectively. The MTT assay results revealed that CMCIP3a displayed a dose-dependent inhibition on five cancer cells (HT29, PC3, HeLa, Jurkat and HepG-2) in the range of 10–320 μg/mL. The APTT, PT and TT were significantly extended by CMCIP3a in relation to dosage, indicating that the anticoagulant effect of CIP was both extrinsic and intrinsic, along with a common coagulation pathway. These findings demonstrated that carboxymethylation might effectively improve the biological potential of the derivatives and offer a theoretical framework for the creation of novel natural antioxidants, low-toxicity antitumor and antithrombotic drugs.

## 1. Introduction

Oxidative stress refers to an imbalance between a biological system’s ability to swiftly detoxify reactive intermediates, also known as reactive oxygen species (ROS), or the harm that results from the widespread manifestation of these species. Among other things, the DNA, lipids and proteins can be disturbed because of the generation of free radicals, which disrupts the normal redox state of the cells. Only a few of the chronic illnesses that might develop as a result of such molecular destruction include atherosclerosis, cancer, diabetes, cardiovascular and respiratory ailments [1]. The body produces antioxidants through various methods to protect against oxidative stress. These processes include endogenous generation of antioxidants in addition to external supply via diet. Antioxidants are substances that quench free radical activity to reduce or prevent cellular damage. However, most often-utilized antioxidants are synthetic and have been connected to liver damage and cancer [2]. In addition, cancer has been one of the leading causes of mortality, posing a threat to humanity. A significant barrier to the unfavourable side-effects and long-term survival of cancer continues to be improved therapeutic efficacy in cancer treatments. Due to their diminished cytotoxic effects, natural antioxidants and cancer preventative medications must be understood and explored in order to assist people in reducing excessive ROS generation and any harm brought on by the malignancy. The cell walls or membrane of plants, fungi and microbes commonly contain polysaccharides, which are naturally occurring antioxidants [3]. The antioxidant capabilities of natural polysaccharides from several medicinal fungi have received more attention lately. Mushroom-derived polysaccharides have drawn a lot of interest due to their important roles as nutritional and medicinal agents [4].

Studies have shown that the biological features of polysaccharides are influenced by their molecular weight (Mw), monosaccharide composition, degree of substitution (DS), glycosidic link, positioning of the branched chains and structure [5]. The bulk of naturally occurring polysaccharides have little bioactivities as a result of their structure and physico-chemical properties. The polysaccharide structure can be changed utilizing physical, chemical and biological methods to address this issue. Carboxymethylation, sulfation, selenylation and acetylation are thought to be efficient methods for increasing the solubility of polysaccharides in water and favourably affecting their bioactivity [6]. The biological activity of the polysaccharide is often enhanced when the hydroxyl group is replaced with the carboxymethyl group present in the polymer chain. The chemical modification of new polysaccharide derivatives and connections between polysaccharide structures and activities have attracted increasing interest because they can enhance the biological effects. Additionally, research on the relationship between polysaccharide structure and activity has demonstrated that most natural polysaccharides’ bioactivities are influenced by their structures either directly or indirectly [7].

The studies focusing on the structural characterization, carboxymethylation and biological characteristics of polysaccharides from edible and medicinal fungi have increased during the past few years. A comparative evaluation of the in vitro antioxidant activities of unmodified and carboxymethylated polysaccharides derived from *Craterellus cornucopioides* [8], *Pholiota nameko* [9], *Ganoderma lucidum* [10], *Tremella fuciformis* [11], *Lachnum* sp. [12], *Folium isatidis* [13], *Sargassum fusiforme* [14] and evaluation of in vitro antitumor activity investigated in a polysaccharide and its carboxymethylated derivative, derived from *Cyclocarya paliurus* [6], *Poria cocos* [15], *Ganoderma lucidum* [10] and *Pleurotus tuber-regium* [16] revealed that carboxymethylation enhanced their biological activity in addition to affecting their physicochemical properties.

India is the home of the edible milky mushroom, *Calocybe indica*, belonging to the family Lycophyllaceae and order Agaricales. In our earlier research, we mainly concentrated on extracting, separating and characterizing the polysaccharides from different mushrooms [17,18,19]. This research has shown that the crude polysaccharides from *C. indica* exhibited various biological properties, including antioxidant, antitumor and anticoagulant activities [20]. The restoration of the antioxidant enzyme activity and lipid peroxidation reduction has helped the *C. indica* polysaccharide in demonstrating significant antioxidative and anti-aging effect in D-galactose-induced aging in mice [21]. To the best of our knowledge, there has been no report on the biological activity of carboxymethylation of the target mushroom polysaccharides. In conformance with the above, the structure of CIP was altered by carboxymethylation. Herein, we isolated a water-soluble polysaccharide using hot water extraction, subsequently purified and carboxymethylated derivatives were prepared. Further, the physicochemical properties, antioxidant activity, antitumor effect and anticoagulating activity were performed through in vitro analyses and compared to the unmodified polysaccharide. The goal of this research was to explore the relationship between structural and biological activity variations, as well as to elucidate any potential processes underlying the antioxidative and anticancer effects.

## 2. Materials and Methods

### 2.1. Reagents

The fresh fruiting bodies of *Calocybe indica* were obtained from a mushroom farm in Coimbatore (Tamil Nadu, India). The following chemicals were purchased from Sigma Aldrich (St. Louis, MO, USA): DEAE cellulose 52, Sepharose 6B, T series dextrans, monosaccharide standards, rhamnose (Rha), arabinose (Ara), xylose (Xyl), galactose (Gal), ribose (Rib), mannose (Man), fucose (Fuc) and glucose (Glc). The APTT, PT and TT reagent kits were procured from Agappe Diagnostic Ltd. (Cochin, India). All other analytical reagent-grade chemicals were acquired from Merck and Himedia in India. A Milli-Q water purification system was used to provide the ultra-pure water (Millipore, Bedford, MA, USA). The National Centre for Cell Science (Maharashtra, India) provided the colon adenocarcinoma (HT29), human cervical cancer cell lines (HeLa), prostate adenocarcinoma cells (PC3), human lymphocytic leukaemia cell-lines (Jurkat) and liver cancer (HepG2) cells.

### 2.2. Isolation and Purification of the Polysaccharide

**Figure 1:**
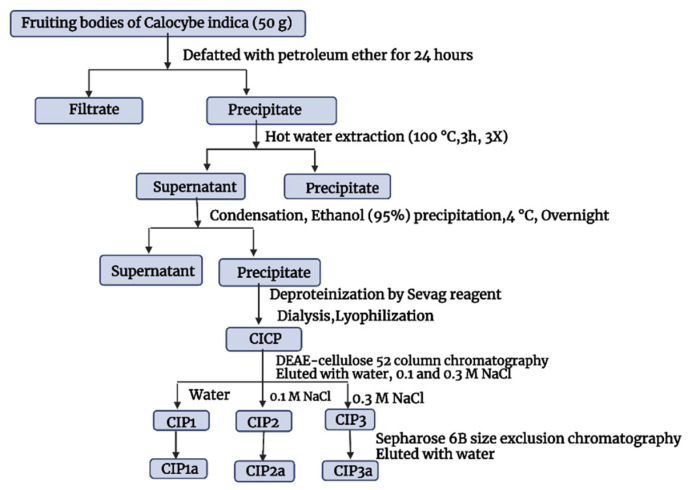
Extraction and purification of *Calocybe indica* polysaccharides.

The crude polysaccharides of *C. indica* (CIP) were extracted using hot water, treated with Sevag to remove protein, precipitated with ethanol in accordance with the procedure we previously described by Govindan et al. [17]. The crude CIP was purified using anion-exchange column chromatography and gel-filtration column chromatography, and this process was checked using the phenol–H_2_SO_4_ technique. The DEAE-Cellulose 52 column (3.0 × 50 cm) was used to fractionate CIP (100 mg), which was dissolved in Millipore water (10 mL). The sample was eluted with a linear gradient sodium chloride solution (0.1 M and 0.3 M) at a flow rate of 1 mL/min (5 mL tube). Elution profiles for the three fractions [CIP1 (neutral polysaccharide), CIP2 and CIP3 (acidic polysaccharide)] were isolated as shown in Figure 1. Using a Sephacryl 6B column (1.0 × 30 cm), the CIP3 was further purified before being eluted with 0.5 mL/min of ultra-pure water. A pure polysaccharide, referred to as CIP3a, was obtained by collecting, concentrating and lyophilizing the peak that eluted.

### 2.3. Carboxymethyl Derivative Preparation of CIP3a

CIP3a was carboxymethylated by combining 300 mg CIP3a with 12.5 mL isopropanol and vigorously stirred for 15 min at room temperature. Successively, 5 mL of 20% NaOH was added dropwise to the reaction mixture. The carboxymethylation mixture (1.37 g chloroacetic acid + 5 mL 20% NaOH + 12.5 mL isopropanol) was added while stirring after 3 h at room temperature. The reaction mixture was then kept at 60 °C for another 4 h. The pH of the reaction mixture was adjusted to 7 with 0.5 M HCl after cooling the mixture to room temperature. The product was dialyzed for 12 h with tap water and then for 48 h with deionized water. The non-dialyzable layer was concentrated and precipitated at 4 °C for 12 h with 95% (*v*/*v*) ethanol. The carboxymethylated derivative obtained was freeze-dried and designated as CMCIP3a. The acid–base titration method was used to determine the degree of substitution (DS) [22]. The following formula was used to determine DS [23]: DS = 162A/(1 − 0.058A); A = (V_0_C_0_ − V_1_C_1_)/m; where A is the millimoles of sodium hydroxide consumed per gram sample, V_0_ is the volume of sodium hydroxide consumed, C_0_ is the concentration of sodium hydroxide, V_1_ is the volume of hydrochloric acid consumed, C_1_ is the concentration of hydrochloric acid and m is the sample weight.

### 2.4. Properties of CIP3a and CMCIP3a

#### 2.4.1. Chemical Composition Analysis

The chemical compositions were determined based on previous research [18]. The phenol–sulfuric acid method was employed in the determination of the total sugar content, with the standard set as D-Glucose. The protein content was evaluated using the Bradford method, setting bovine serum albumin as the standard. The carbazole–sulfuric acid technique was used to determine uronic acid concentration, having glucuronic acid as the reference. The solubility in water was determined following the procedure described by Liu et al. [24], and the results were expressed as the weight of polysaccharide per mL of water (mg/mL).

#### 2.4.2. Spectral Analyses

On a spectrophotometer (UV-1800, Shimadzu Kyoto, Japan), the UV-Vis absorption spectra of CIP3a (0.5 mg/mL) and CMCIP3a (0.5 mg/mL) were obtained with wavelength ranges set between 200 and 500 nm. The infrared spectrum was captured at room temperature using an FT-IR spectrometer (Thermo-Scientific Nicolet, 5700IR Waltham, MA, USA). The polysaccharides were pressed into pellets with KBr powder for infrared spectral collection in the 400–4000 cm^−1^ range. On a 500 MHz NMR spectrometer (Bruker AVII 500 MHz Germany), the ^1^H and ^13^C NMR spectra of the CIP and CMCIP were captured. The samples were dissolved in the heavy water.

#### 2.4.3. Molecular Weight and Monosaccharide Composition Analysis

Following an approach presented in our previous study [17], high-performance size-exclusion chromatography and a Shodex SB-804 HQ column equipped with a refractive index detector (RID) were used to measure the molecular weight of CIPs. Each run involved the injection of a 10 µL sample (2 mg/mL) at a flow rate of 0.9 mL/min with a column temperature of 45 °C. By correlating the retention time of a standard curve made using a number of dextran standards, the molecular weight was determined. The monosaccharide content of CIPs was assessed using 1-phenyl-3-methyl-5-pyrazolone (PMP) derivation and HPLC (Agilent, Santa Clara, CA, USA) analysis as outlined in our previous research [17]. The Agilent system with a Zorbax Eclipse Plus C18 column (5 μm, 4.6 mm × 250 mm) operating at 30 °C served as the HPLC system. The mixture of 83% of 0.05 M phosphate buffer solution (pH 6.7) and 17% of acetonitrile were use as mobile phase. The injection volume was 5 µL and the flow rate was 0.7 mL/min. To get chromatograms, the diode array detector was tuned at 254 nm. As references, mannose, ribose, rhamnose, glucose, galactose, xylose, arabinose, fucose, glucuronic acid and galacturonic acid were used.

#### 2.4.4. XRD Analysis

The crystal structure of CIP3a and CMCIP3a were identified using an X-ray diffractometer (Siemens D 5000, Bruker, Germany), with 2θ ranging from 10° to 70° at room temperature The time between each step and the step size were 0.01 and 0.1 s, respectively.

#### 2.4.5. Morphological Analysis

Prior to observation, dried CIP3a and CMCIP3a were sputter coated with a gold layer, and images were captured using a high-resolution scanning electron microscope (FEI-Quanta 200 FEG) at a voltage of 5–10 kV and a magnification of 1000–20,000×.

#### 2.4.6. Congo Red Analysis

The Congo red technique was used to determine the triple helical structure of CIPs according to the protocol reported by Natraj et al. [20]. Briefly, 2 mL of Congo red (100 µM) was combined with CIP3a and CMCIP3a (6 mg/2 mL), and the mixture was then bonded at various NaOH concentrations (0.1–0.5 M). Congo red solution without sample was used as control. The maximal absorption wavelength was determined using an ultraviolet-visible spectrophotometer between 400 and 600 nm.

### 2.5. In Vitro Antioxidant Activity

The DPPH, ABTS, hydroxyl and superoxide anion radical scavenging activity, ferric and copper reducing capabilities, and erythrocyte hemolysis inhibition were evaluated using previously reported methods [7,25,26].

### 2.6. In Vitro Antitumor Activity

CIPs cytotoxicity was evaluated following the methodology previously described by Mosmann [27]. On DMEM media (10% foetal bovine serum and 1% penicillin + streptomycin), cancer cells (HT29, PC3, HeLa, Jurkat and HepG2) were grown. In 96-well microplates, 5 × 10^3^ cells/mL were grown overnight at 37 °C and 5% oxygen before being pre-treated for 24 h with a series of doses of CIP3a and CMCIP3a (10–320 µg/mL) and the positive drug cisplatin. The media was then removed after the cells were stained with 20 µL MTT at a concentration of 5 mg/mL in phosphate-buffered saline for 4 h in the dark. After 1 h at 37 °C in which the cells were treated with formazan crystals that had been dissolved in DMSO, the absorbance values were read at 570 nm using a Bio Tek ELISA microplate reader (Winooski, VT, USA). The formula for calculating the anti-proliferative activity (%) was [(Ac-As)/(Ac-Ab)] × 100, where Ac, As and Ab stand for the absorbances of the reference, the sample and the blank, respectively.

### 2.7. Anticoagulant Activity

A semi-automatic hemagglutinator (Transasia Bio-Medicals Ltd., Mumbai, India) was used to determine the anticoagulant activity of CIP3a and CMCIP3a (5 and 10 mg/mL dissolved in saline). The stated procedure involved the use of specialized reagents and standard human plasma to determine in vitro activated partial thromboplastin time (APTT), thrombin time (TT) and prothrombin time (PT) [28].

### 2.8. Data Analysis

The mean and SD of three replicates were used to express the data. An analysis of variance (ANOVA) was utilized using the statistical package SPSS19.0 (IBM corporation, Armonk, NY, USA). Duncan’s multiple range test (DMRT) was used to calculate the differences in the means. All values with a *p* < 0.05 were considered statistically significant.

## 3. Results and Discussion

### 3.1. Carboxymethylation and Degree of Substitution

**Figure 2:**
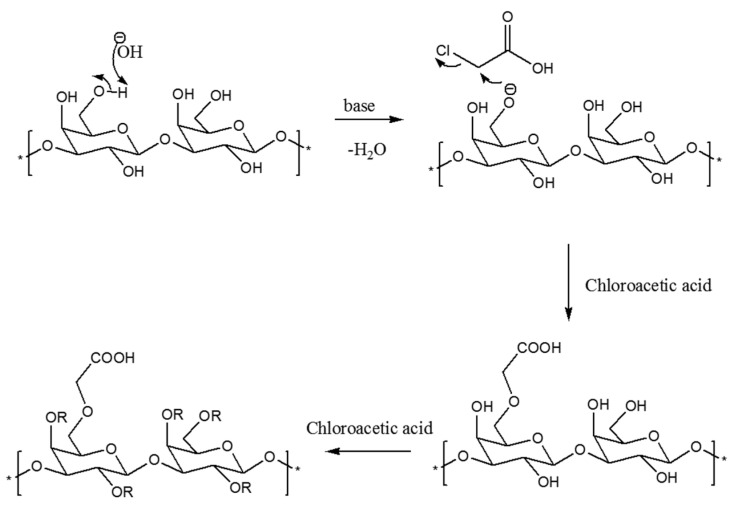
Mechanism of carboxymethylation reaction.

The CMCIP3a were formed by reacting CIP3a with chloroacetic acid in the presence of sodium hydroxide. The CIP3a underwent a two-step etherification process during carboxymethylation. The Williamson’s ether synthesis, a bimolecular nucleophilic substitution mechanism, formed the basis of the carboxymethylation reaction process (S_N_2) [11]. First, the reaction between sodium hydroxide and the hydroxyl groups on the CIP3a led to the formation of the alkoxide groups. Second, the S_N_2 reaction produced carboxymethyl groups between the CIP3a alkoxide and monochloroacetic acid (Figure 2). However, when sodium monochloroacetic and sodium hydroxide were combined to produce sodium glycolate, a side reaction could occur [29]. The success of carboxymethylation was usually assessed using DS, since one of the components that can alter the structure and biological activity of a polysaccharide is DS. The quantitative measurement of the carboxymethyl groups used the DS measured using neutralization titration to obtain the degree of carboxymethylation against the substituted hydroxyl group. Table 1 shows that the DS of CIP3a was 0.345, which was consistent with the prior findings on the *Tremella fuciformis* polysaccharide [11]. 

### 3.2. Isolation and Purification of the Polysaccharide

**Figure 3:**
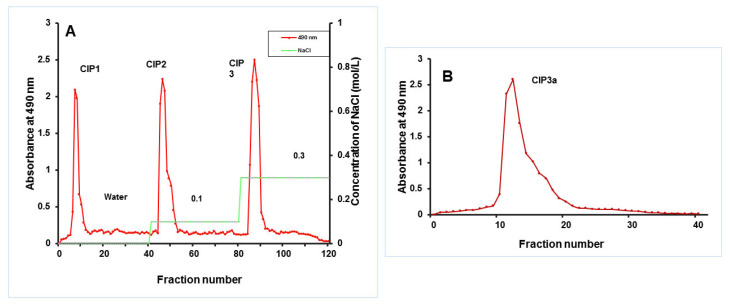
(**A**) Elution profiles of crude CIP on a column of DEAE-cellulose, obtaining fractions CIP1, CIP2 and CIP3; (**B**) Elution profile of CIP3a on Sephacryl 6B column.

Following the extraction with hot water, ethanol precipitation, Sevag’s deproteinization and lyophilization, the crude polysaccharide (CIP) was obtained (Figure 3A). On a DEAE-52 cellulose column, the CIPs were separated and CIP1 was eluted with deionized water, CIP2 with 0.1 M NaCl and CIP3 with 0.3 M NaCl (Figure 3A). With the aid of gel permeation chromatography, using Sepharose 6B and ultrapure water, CIP3a was further fractionated with higher levels of carbohydrate and uronic acid. The eluent was then concentrated, dialyzed and lyophilized. The single elution peak CIP3a (Figure 3B) was further carboxymethylated and denoted as CMCIP3a.

### 3.3. Chemical Composition

The yield, total sugar, protein and uronic acid content in the CIP3a and CMCIP3a polysaccharide extracts are displayed in Table 1. CMCIP produced a yield of 39.15%. CMCIP3a demonstrated a considerable shift in chemical composition compared to native CIP3a. After carboxymethyl alteration, there was a reduction in the total sugar and protein content. In general, the important physicochemical marker, neutral sugars, was believed to have decreased because of the addition of functional groups, which produced higher Mw [30]. CMCIP3a had a higher uronic acid content than CIP3a due to the hydrolysis of CIP3a, which could raise the uronic acid level [31]. In alkaline conditions, the β-elimination process during carboxymethylation could result in an obvious distinction between CMCIP3a and CIP3a. Similar results of reduced total sugar, protein and increase in uronic acid contents were observed in previous studies with *Cyclocarya paliurus* [6] and *Amana edulis* polysaccharide [23], and could be attributed to the carboxymethyl group. In agreement with Xu et al. [10], CMCIP3a had a higher water solubility compared to CIP3a, which may be attributed to an increase in the hydrophilic carboxyl groups.

### 3.4. Molecular Weight and Monosaccharide Composition

**Figure 4:**
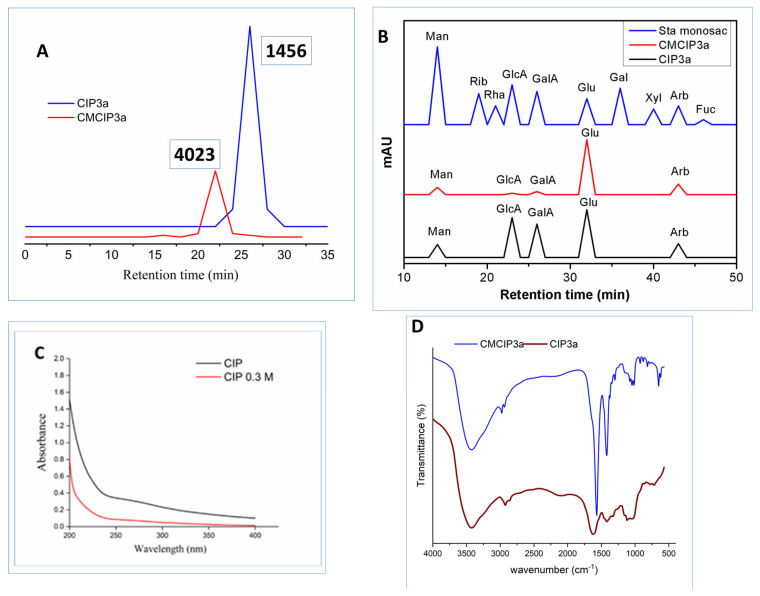
(**A**) HPGPC analysis of CIP3a and CMCIP3a, (**B**) monosaccharide composition of CIP3a and CMCIP3a, (**C**) UV–vis spectrum of CIP3a and CMCIP3a and (**D**) FT-IR spectrum of CIP3a and CMCIP3a.

The molecular weight of CIP3a and CMCIP3a was determined using HPGPC. Retention time was used to compute the Mw of CIP3a and CMCIP3a. In the HPGPC spectra, only a single symmetrical narrow peak could be seen, showing that CIP was obtained as a homogenous polysaccharide, as shown in Figure 4A. The average molecular weights of CIP3a and CMCIP3a were 1.456 × 10^3^ and 4.023 × 10^3^ Da, respectively, which was determined using a series of dextrans with known molecular weights. The Mw of CMCIP3a rose in comparison to CIP3a, and the rise in Mw was 63.81% more than CIP3a, indicating that the derivatives were successfully changed without degradation. Similar results were also reported for the Mw of carboxymethylated polysaccharides derived from *Cyclocarya paliurus* [6] and blackcurrant fruits [7], which were higher than those of native polysaccharides.

CIP3a and CMCIP3a were hetero-polysaccharides, which had molar percentages of Ara, Man, Glc, GlcA and GalA of 18.61, 17.31, 61.56, 0.45, 2.03 in CIP3a and 15.27, 11.05, 70.37, 0.56, 2.75 in CMCIP3a, respectively (Table 1 and Figure 4B). Glc predominated among the monosaccharides, demonstrating that they formed the structural basis of the polysaccharide. CMCIP3a showed a higher molar percentage of Glu, GlcA, GalA and a lower molar percentage of Arb and Man compared to CIP3a. Similar findings have been obtained by carboxymethylation of the polysaccharides of *Cyclocarya paliurus* [6]. CIP3a and CMCIP3a included the same three monosaccharides at different molar percentages, which was consistent with the findings found in blackcurrant polysaccharides and their carboxymethylated derivatives containing the same five monosaccharides at various molar ratios [7]. These findings demonstrated that the addition of functional groups might not have an impact on the polysaccharide’s backbone structures, but it could modify the proportion of the monosaccharides within the polysaccharide.

### 3.5. UV Analysis

Figure 4C displays the UV-vis spectra of CIP3a and CMCIP3a in the 190 to 400 nm wavelength range. CIP3a and CMCIP3a showed no discernible changes in the UV-vis spectra. The peaks that occurred between 260 and 280 nm showed that protein and nucleic acids were present in trace amounts, which was in line with chemical composition analysis. 

### 3.6. FTIR Analysis

The FT-IR spectra of CIP3a and CMCIP3a showed comparable polysaccharide absorbance peaks in the spectra obtained between 4000 cm^−1^ and 400 cm^−1^ (Figure 4D). However, some variations in a particular spectral range may be seen. OH stretching vibrations were represented by the band at 3424/3424 cm^−1^, while a CH stretching peak attributable to the CH_2_ groups was observed at 2977/2924 cm^−1^ [32]. Carbohydrates showed typical absorbance peaks between 1200 cm^−1^ and 800 cm^−1^. Furthermore, three new notable peaks were identified at 1625 cm^−1^, 1420 cm^−1^ and 1328 cm^−1^, which corresponded to asymmetric and symmetric COO- stretching vibrations, respectively, showing that the carboxymethylation of CIP3a was successful [33,34]. 

### 3.7. NMR Analysis

The polysaccharide was subject to ^1^H and ^13^C NMR analyses to characterize the carboxymethylated product (Supplementary Materials Figure S1). The solvent used was deuterium oxide (D_2_O), which exhibited a signal at 4.701 ppm in corroboration with the available reference data (4.800 ppm). The characterizing signal to observe in the case of the ^1^H NMR was the signal that was produced by the methylene group of the carboxymethyl group introduced in the reaction. Hydrogens within this group portrayed a characteristic signal of approximately 3.9–4.2 ppm. The ^13^C NMR analysis was performed in tandem with the ^1^H NMR analysis to aid in the confirmation of the formation of the desired product. The literature shows that carbonyl carbon appears about 180 ppm, which in our case was observed at 179.39 ppm. This signal confirms the formation of the desired product.

### 3.8. XRD Analysis

The polysaccharides’ nature was frequently determined via XRD analysis. The crystal structure of the polysaccharides directly influences their physical characteristics, such as tensile strength, expansibility, solubility, flexibility and others. Figure S2 displays the XRD patterns of the CIP3a and CMCIP3a. For CIP3a, there was a strong and sharp diffraction peak at the 2θ of 32° and weak peaks at the 2θ of 45°, 57°, 66° and 76°. After carboxymethylation, the XRD properties of CMCIP3a were very different from those of the natural polysaccharide and showed amorphous structure in the form of a halo peak near 2θ of 20° and 25°. Therefore, structural derivatization had a significant impact on the crystalline structure of the polysaccharides since CIP3a and CMCIP3a had distinct molecular structures. The findings corroborated earlier observations that the native and carboxymethylated forms of the *Folium isatidis* polysaccharide had crystalline or amorphous structures, respectively [13].

### 3.9. Morphological Analysis

**Figure 5:**
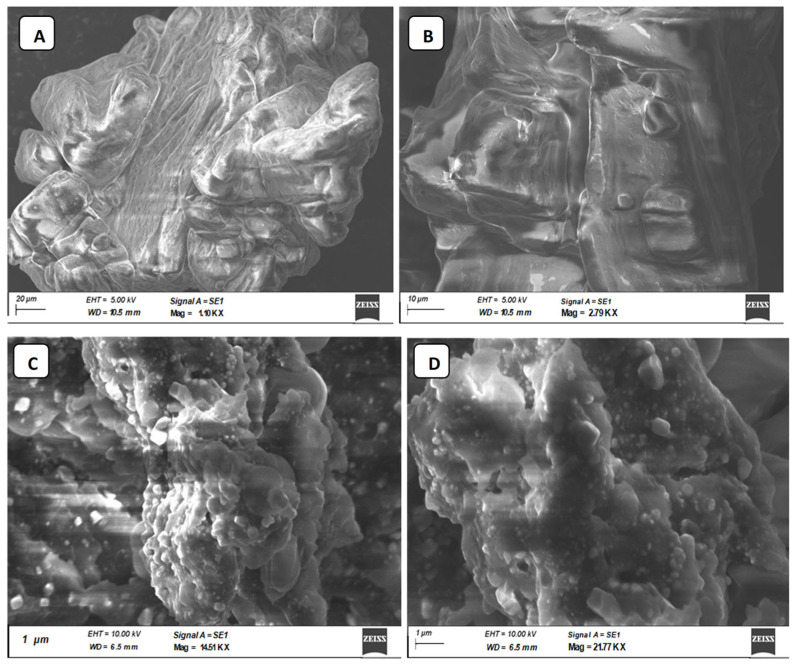
Photomicrographs of CIP3a and CMCIP3a recorded by SEM. (**A**) 1.10 KX × CIP3a, (**B**) 2.79 KX × CMCIP3a, (**C**) 14.51 KX × CMCIP3a, (**D**) 21.77KX × CMCIP3a.

The SEM images were used to directly demonstrate the chain conformations of CIP3a and CMCIP3a to elucidate their physical microstructure (Figure 5). Figure 5A depicts the SEM image before carboxymethylation and Figure 5B portrays the SEM image after the process. Smooth-surfaced spherical formations could be seen at magnifications of up to 2000× (Figure 5A). Following carboxymethylation, the looseness of the structure was seen, along with a rough and porous surface and a bigger particle, suggesting that there may be a weaker contact between the CMCIP3a particles. 

### 3.10. Congo Red Analysis

**Figure 6:**
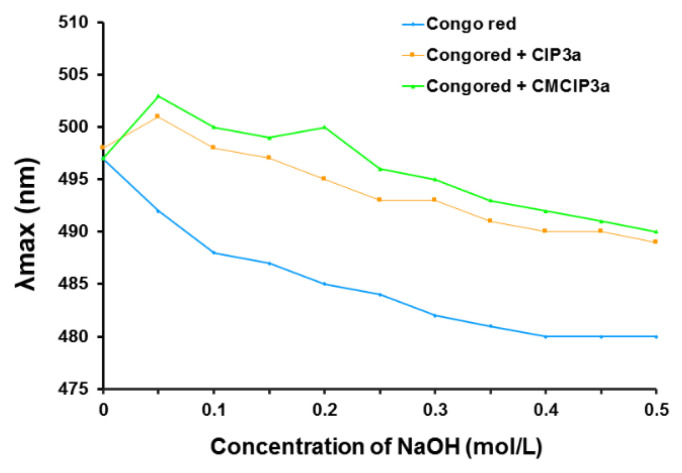
Effect of polysaccharide on the absorbance of Congo red.

Congo red dye and polysaccharides with triple helical conformations can combine in an alkaline environment, forming a complex that exhibits a bathochromic shift in the maximum absorption wavelength of the Congo red solution. The maximum absorption wavelength of Congo red + CIP3a and Congo red + CMCIP3a was notably higher than that of the control group (Congo red) (Figure 6), which would be due to an increase in the concentration of the NaOH solution that increased from 0 to 0.5 mol/L, demonstrating that CIP3a and CMCIP3a had triple helical structures.

### 3.11. Antioxidant Activity of CIP3a and CMCIP3a

Antioxidant activities can be attributed to a variety of processes and reactions, such as radical scavenging activity, binding of transition metal ion catalysts and chain initiation prevention. In the current study, the antioxidant properties of CIP3a and CMCIP3a were assessed in terms of DPPH, ABTS, superoxide, hydroxyl radical scavenging activity, iron and copper reducing power ability and erythrocyte hemolysis inhibition.

#### 3.11.1. DPPH Radical Scavenging Activity 

**Figure 7:**
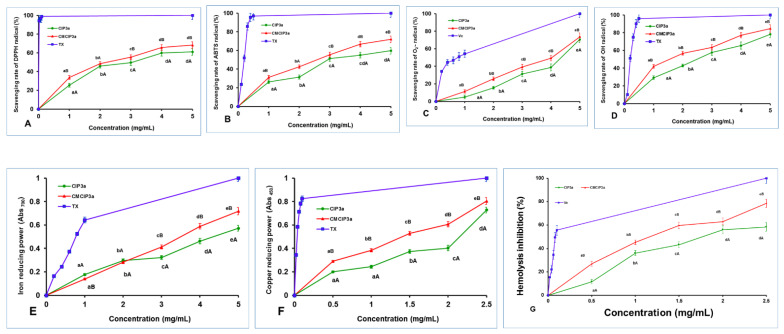
Antioxidant activity of CIP3a and CMCIP3a. (**A**) DPPH radical scavenging, (**B**) ABTS radical scavenging, (**C**) superoxide radical scavenging, (**D**) hydroxyl radical scavenging, (**E**) ferric ion reducing power, (**F**) cupric ion reducing power and (**G**) erythrocyte hemolysis inhibition. Data are the mean ± SD of three replicates, and small letters in superscripts (a–e) and capital letters (A,B) indicate significant differences (*p* < 0.05) between concentrations of the same sample and different samples of the same concentration, respectively.

The DPPH radical exhibits its distinctive purple colour at 517 nm, and when antioxidants are added, this colour tends to diminish [35]. This study used ascorbic acid as a positive control to test the DPPH-scavenging abilities of CIP3a and CMCIP3a. Both CIP3a and its derivative (CMCIP3a) were shown to have DPPH scavenging activity in a dose-dependent manner, as shown in Figure 7A. 

The scavenging ability of both samples rose with increasing concentration in the range of 1–5 mg/mL, reaching their maximum values of 61.06% and 68.29% at 5 mg/mL, respectively. The half maximal effective concentrations (EC_50_) of CIP3a and CMCIP3a were 3.29 and 2.49 mg/mL, respectively, whereas Trolox showed a value of 0.022 mg/mL. Therefore, considering the EC_50_ value, the scavenging activities of CMCIP were superior to those of CIP3a, but lower than those of Trolox. At every concentration, the DPPH scavenging activity of CIP3a was observed to be weaker, whereas the ability of CMCIP3a was found to be stronger. These findings were in line with those reported by other researchers [36,37]. The above findings suggest that the carboxymethylated derivatives, particularly at high concentrations, have an appreciable impact on the scavenging of DPPH and can be used as natural antioxidants.

#### 3.11.2. ABTS Decolourization Capacity

The ABTS method, which is extensively used to assess antioxidant capability, works on the principle that when blue-green ABTS•+ is combined with antioxidants, the solution turns colourless [38]. The scavenging activities of CMCIP3a (2.66 mg/mL) were shown to be better than those of CIP3a (2.98 mg/mL), although both were less effective than Trolox (0.193 mg/mL) when the EC_50_ value was accounted for. The ABTS radical cation inhibition of CIP3a and CMCIP3a at 5.0 mg/mL was 59.71% and 72.01%, respectively (Figure 7B). The ABTS radical scavenging activity of CIP3a was lowest and that of CCIP3a was the highest at all concentrations tested. These results were consistent with those reported in other studies [36,37]. These findings suggest that the carboxymethylated derivative should be explored as a promising antioxidant because it has a potent capacity to capture the ABTS radical.

#### 3.11.3. Superoxide Radical Scavenging Activity

The self-oxidation of pyrogallol is a chain reaction that can yield O_2_^•−^ and a spectrophotometer set to 325 nm detects the reaction. Under alkaline environments, the auto-oxidation rate of pyrogallol is proportional to the concentration of releasing O_2_^•−^. As a result, assessing the scavenging effect of antioxidants on O_2_^•−^ in the system can be used to indirectly assess their antioxidant capacity [39]. As demonstrated in Figure 7C, the superoxide scavenging capacities of CIP3a, CMCIP3a and vitamin C rose significantly with increasing sample concentration (1–5 mg/mL). The superoxide radical anion inhibition of CIP3a and CMCIP3a at dosages of 1 to 5 mg/mL ranged from 5.01 to 70.62% and 11.42 to 73.57%, respectively. The EC_50_ values for CIP, CMCIP and vitamin C were 4.52, 4.10 and 0.76 mg/mL, respectively. Carboxymethyl groups are potent electron-withdrawing groups, and carboxymethyl modification improves the superoxide anion’s ability to accept hydrogen as a donor, as well as the activity of radial scavengers. In contrast to non-carboxymethylated garlic polysaccharides, Cheng and Huang [40] found that carboxymethylated garlic polysaccharide performed effectively against superoxide anion radical antioxidant activity.

#### 3.11.4. Hydroxyl Radical Scavenging Activity

The hydroxyl radical is considered as a very powerful oxidant, since it can easily penetrate cell membranes, harm tissue and kill cells. In light of this, eliminating hydroxyl radicals is crucial in determining antioxidant protection in dietary or cellular systems. Numerous research studies have focused on reducing the production of hydroxyl radicals or cleaning up already-produced hydroxyl radicals [41,42]. A reaction between hydroxyl radicals and a FeSO_4_ solution was triggered by the addition of H_2_O_2_. The salicylic acid then undergoes a reaction with the hydroxyl radicals. The concentration of hydroxyl radicals had a dose–effect relationship with the coloured substance that resulted in terms of its absorbance at 510 nm. Figure 7D shows that raising the concentration of CIP3a and CMCIP3a increased their ability to scavenge OH radicals, with CMCIP3a having the strongest ability to do so. At 5 mg/mL, the effects of CIP3a and CMCIP3a were 78.17% and 84.60%, respectively. According to the EC_50_, the scavenging effect of CMCIP3a (1.60 mg/mL) against OH was much higher than that of CIP3a (2.61 mg/mL), but weaker than Trolox (0.23 mg/mL). These findings suggested that carboxymethylation of CIP improved its OH scavenging activity substantially at every concentration. The *Auricularia auricula* polysaccharide (AAP) had a 27.75% OH scavenging efficiency at 2 mg/mL compared to 52.13% for the carboxymethylated AAP (CMAAP). The OH scavenging capacities of CIP3a and CMCIP3a were 42.89% and 56.77%, respectively, showing that CIPs were more effective than CMCIP3a in removing hydroxy radicals [43]. The significantly strong scavenging activity of OH radicals may be caused by the fact that the carboxymethyl groups’ introduction of methylene hydrogens tended to remove the reactive OH radicals in order to stop the radical chain reaction. Therefore, the two suggested mechanisms for removing hydroxyl radicals are as follows: the first involves chelating the sample with a transition metal ion that can activate H_2_O_2_, preventing the production of hydroxyl radicals; the second consists of reducing the hydroxyl radical by introducing hydrogen ions into the sample being examined [10].

#### 3.11.5. Ferric Reducing Power Ability

Since antioxidants prohibit the reduction of the Fe^3+^ ion within the ferricyanide complex to the Fe^2+^ form, which is assessed by measuring the intensity of the characteristic Prussian blue colour at 700 nm, ferric reducing assay is a good predictor of antioxidant activity [44]. As displayed in Figure 7E, there were significant differences between CIP3a and CMCIP3a at each concentration tested, and their reducing power rose linearly with the concentration between 1 mg/mL and 5 mg/mL. The absorbance of CIP3a at 5 mg/mL was 0.572 but that of CMCIP3a was 0.718, indicating that CMCIP3a exhibited a significant reducing power because of its lower Mw. According to the EC_50_ value, the sequence of the reducing power was Trolox (0.78 mg/mL) > CMCIP3a (3.48 mg/mL) > CIP3a (4.39 mg/mL). In comparison with the native polysaccharide CIP3a, CMCIP3a, with little substitution, had a somewhat higher reducing power, which was in agreement with previous research [45]. The presence of reductones, molecules that break free radical chains by donation of a hydrogen atom, has previously been linked to the presence of reducing characteristics [46]. It has also been reported that reductones react with peroxide precursors, inhibiting the generation of the peroxide species. However, numerous studies have demonstrated that a polysaccharide’s biological activity is determined by solubility, composition of the polysaccharide, along with structural features such as Mw, configuration and so on. In this assay, the reducing power of CIP3a increased with increasing concentrations, whereas the carboxymethyl groups reduced the hydroxyl groups and changed its steric conformation, lowering the electron density on the active hydroxyl groups and preventing some active carboxymethyl groups from binding to the metal ion. Several studies have revealed that the existence of uronic acid residues, a stable triple helical structure, the inclusion of DS and the protein content of the polysaccharide were key aspects for lowering the production of hydroxy radicals and enhancing the reducing power of the polysaccharide [47].

#### 3.11.6. Copper Reducing Ability

The CUPRAC assay involves a redox reduction between the antioxidants and the CUPRAC reagent when the sample contains a leading thiol group. In this procedure, the reagent self-reduces to produce a copper (I)-neocuproine chelate complex, which produces a colour detectable at 450 nm. As sample concentration increased, the cupric reducing power of CIP3a and CMCIP3a increased as well (Figure 7F). CIP3a had an absorption of 0.728 at 2.5 mg/mL and an EC_50_ of 2.08 mg/mL, whereas CMCIP3a had an absorption of 0.804 at the same dose and an EC_50_ of 1.41 mg/mL. The reducing power of CMCIP3a was significantly higher than that observed for CIP, although both were inferior to Trolox (EC_50_ = 0.033 mg/mL). CIP3a and CMCIP3a may function as electron donors, transferring electrons to free radicals and transforming them into more stable molecules, in conformity with the results of CUPRAC. 

#### 3.11.7. Erythrocyte Hemolysis Inhibition

The most prevalent blood cells that deliver oxygen to human tissues via the blood are erythrocytes. Because of the high amount of oxygen, hemoglobin and membrane polyunsaturated fatty acids, erythrocytes are particularly vulnerable to oxidative damage [48]. As an essential reactive oxygen species (ROS), hydrogen peroxide (H_2_O_2_) can easily traverse the cell membrane and react with Hb to produce reactive radical species, which eventually causes oxidative stress on the erythrocyte membrane [49]. H_2_O_2_ was chosen as the free radical initiator in this investigation because it might eventually lead to the hemolysis of erythrocytes, and hemolysis inhibition is an indirect technique that assesses the intracellular antioxidant capacity. As a result, H_2_O_2_ is frequently employed to create oxidative stress in vitro in order to assess erythrocyte oxidative damage. The protective effects of CIP3a, CMCIP3a and Vc against H_2_O_2_-induced erythrocyte hemolysis were concentration-dependent, as shown in Figure 7G. The inhibitory rate of CIP3a and CMCIP3a rose from 11.60% to 58.45% (EC_50_ = 3.78 mg/mL) and from 26.99% to 78.57% (EC_50_ = 2.30 mg/mL) as the tested concentrations increased (0.5–2.5 mg/mL). These results demonstrated that CMCIP3a had higher erythrocyte hemolysis protection than CIP3a; however, Vc had lower inhibition rates (EC_50_ = 0.039 mg/mL). Native polysaccharide (RNP) and carboxymethylated polysaccharide (CRNP-1, CRNP-2, and CRNP-3) isolated from blackcurrant fruits had inhibitory rates of 50.95%, 54.75%, 57.12% and 63.69%, respectively, at 4 mg/mL [7].

### 3.12. Inhibitory Effects of CIP3a and CMCIP3a on Tumor Cells

**Figure 8:**
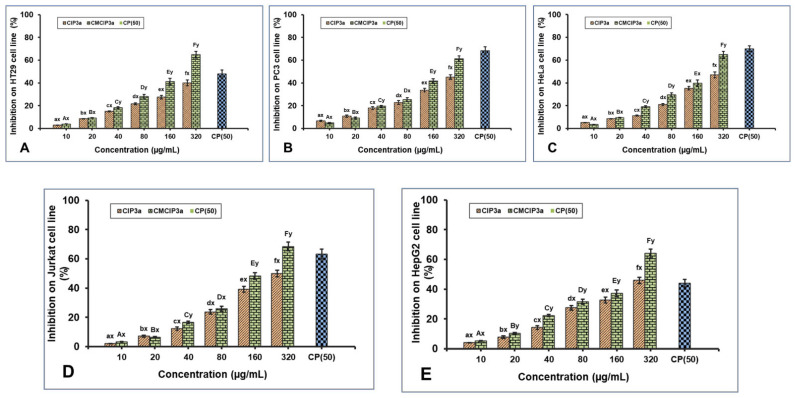
Antiproliferative effects of CIP3a and CMCIP3a on: (**A**) HT29, (**B**) PC3, (**C**) HeLa, (**D**) Jurkat and (**E**) HepG2 cells. Statistically significant (*p* < 0.05) increase in CIP3a and CMCIP3a concentrations are denoted by small (a–f) and capital letters (A–F), respectively. Significant (*p* < 0.05) differences between samples with same concentrations and different ones are denoted by small letters (x, y).

According to earlier studies, polysaccharides can be modified with carboxymethylation to increase their antitumor activity. The antiproliferative effect of CIP3a and CMCIP3a on HT29, PC3, HeLa, Jurkat and HepG2 cells was examined in vitro in the current work. For comparison, cisplatin (CP) at a concentration of 50 µg/mL was used (Figure 8A–E). The outcomes demonstrated a dose-dependent suppression of five cancer cells by CIP3a and CMCIP3a in the range of 10–320 µg/mL. The growth inhibition of CIP3a on HT29, PC3, HeLa, Jurkat and HepG2 cells at 320 µg/mL were 39.97%, 49.10%, 47.03%, 50.01% and 45.84%, respectively. The growth inhibition of CMCIP3a on HT29, PC3, HeLa, Jurkat and HepG2 cells at 320 µg/mL were 63.09%, 61.24%, 64.81%, 68.41% and 64.00%, respectively. Both samples exhibited a considerable degree of tumor cell growth inhibition, and samples at high doses were found to have stronger antitumor effects. It should be highlighted that CMCIP3a demonstrated significantly higher antitumor activity in vitro than CIP3a for growth inhibition at concentrations from 10 to 320 µg/mL. Cisplatin showed a cytotoxicity of 47.86%, 68.28%, 69.93%, 63.32% and 43.98% at 50 µg/mL for HT29, PC3, HeLa, Jurkat and HepG2 cells, respectively. 

In contrast to CIP3a with lower Mw, CMCIP3a with relatively greater Mw exhibited a higher inhibition rate, indicating that the relative increase in Mw increased antitumor activity. This indicated that the addition of ionic groups to the native polysaccharides improved their antitumor properties. The improvement of the antitumor activity of CIP3a depended on several factors, including its high Mw, protein content, expanded chain and good water solubility. This is explained by the fact that the polysaccharides had more opportunities to bind with tumor cell receptors due to the presence of ionic groups or relatively long molecular chains. In the A375, HepG2 and Caco-2 cells, the *Cyclocarya paliurus* polysaccharides (CP) and their carboxymethylated derivative (CM–CP) exhibited various antitumor activities [6]. In contrast, the pure carboxymethylated polysaccharide CMP33 from the *Poria cocos* mushroom significantly reduced the proliferation of HT-29 (84%) and SGC-7901 (96%) cells at 1000 µg/mL [50]. According to reports, carboxymethylated corn bran polysaccharides significantly inhibited the growth of A549 and HepG-2 cells in vitro [32]. Furthermore, *Ganoderma lucidum* (CM-GL) carboxymethylated polysaccharide demonstrated in vitro antiproliferation against S-180 cancer cells with an IC_50_ of 38 µg/mL [51]. In view of the results, the carboxymethyl modification results in novel biological properties, which opens up new possibilities for the use of polysaccharides.

### 3.13. Anticoagulation Activity of CIP and CMCIP

To compare the effects of CIP3a and CMCIP3a on the APTT, PT and TT of normal human plasma to heparin, the anticoagulant activities of CIP3a and CMCIP3a were examined in vitro. These three indicators (APTT, PT and TT) express the intrinsic, extrinsic and common pathways of blood coagulation, respectively. 

**Figure 9:**
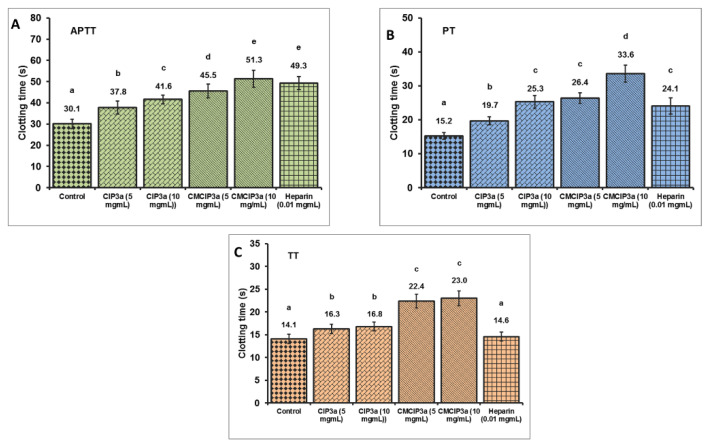
In Vitro anticoagulant properties of CIP3a and CMCIP3a. Values with no letters in common in each clotting test (**A**–**C**) (APTT, PT and TT) are significantly different (*p* < 0.05).

According to the findings shown in Figure 9, a dose-dependent increase in coagulation times was observed in CIP3a for APTT, PT and TT, suggesting an anticoagulant effect via the endogenous, exogenous and common coagulation pathways. Moreover, a dose-dependent increase in the APTT, PT and TT was observed in the case of CMCIP3a. Furthermore, APTT, PT and TT showed that CMCIP3a had much greater anticoagulant action when compared to CIP3a. Even at prolonged APTT and TT concentrations, no appreciable PT lengthening was observed. These findings suggest that the blood coagulation intrinsic, extrinsic and common pathways were used by CIP3a and its derivatives CMCIP3a to execute an anticoagulant effect. The anticoagulant properties of the carboxymethylated *Sepia esculenta* ink polysaccharide are supported by our findings [52].

### 3.14. Relationship between the Biological Effects and Chemical Modification of CIP

Polysaccharides can be chemically modified to produce new antioxidants, antitumor and antithrombotic agents. One of the frequently used chemical modification techniques is carboxymethylation, which involves adding carboxymethyl groups to the hydroxyl groups of polysaccharides. This results in modifications in the physicochemical properties, conformations and primary structures of the polysaccharide [53]. Numerous earlier studies suggested that carboxymethylation boosts the water solubility of natural polysaccharides and enhances the hydrophilicity of polysaccharides, leading to higher water solubility and significantly greater bioactivities. The interactions with positively charged biomolecules rise as the polysaccharide’s electron cloud density and electron-withdrawing activity increase. The biological function of polysaccharide derivatives can also be influenced by additional structural characteristics, such as DS and microstructure [54]. The flexibility of the chain and the spatial conformation of the carboxymethylated polysaccharide’s molecular structure significantly affect its bioactivities. According to Sun et al. [55], the molecular and structural alterations of the polysaccharide may alter its antitumor activity, which is consistent with the findings of the current investigation.

The current findings demonstrated that the in vitro antioxidant activity of CMCIP3a increased significantly. According to a theory, the negatively charged hydrophilic surface structure of the polysaccharide can be created by the carboxymethylation alteration, which considerably improves the polysaccharide’s water solubility and thus increases its antioxidant activity [54]. Additionally, the carboxylate groups within the uronic acid entity in the polysaccharides may operate as hydrogen-donating and electron-transfer agents; acidic polysaccharides are more likely to act as secondary antioxidants [56]. This study found better antioxidant effects in the polysaccharides with higher Mw, which was consistent with other studies [8]. Strong antioxidant action in carboxymethyl derivatives is probably caused by the presence of substitution groups that change the structure of CIP3a and decrease intermolecular/intramolecular hydrogen bonding. The substitution of the carboxymethyl groups dramatically enhanced the polysaccharide’s solubility in water and simultaneously increased the antioxidant, anticancer and anticoagulant properties. It is interesting to note that some research suggests that polysaccharides with higher glucose content may be associated with higher antioxidant activity. Following the modification, it was observed that the amount of glucose within the CMCIP3a and the corresponding antioxidant and antitumor activity were linearly correlated, showing that the ratio of monosaccharide composition to polysaccharide composition also significantly affects the biological function of the polysaccharides. The microstructure of the polysaccharides can also influence the biological activity [12]; indeed, in our study the chemical alteration dramatically changed the microstructure of the CIP3a, as seen in the SEM results. 

Therefore, the findings of our research showed that the antioxidant, anticancer and anticoagulant properties of the CIP3a molecule may be drastically changed by the addition of a carboxymethyl group. In general, the complex structure and structural variability of polysaccharides make it difficult to properly define the relationship between polysaccharide structure and biological activity, and additional research will be needed to determine which components play a predominant role.

## 4. Conclusions

In summary, chemical modification was successful in generating the carboxymethylated derivative of CIP3a, which has a DS of 0.345. The valuable information provided by different analytical methods showed that CMCIP3a have possessed quite different physicochemical and structural properties. CIP3a and CMCIP3a are both hetero-polysaccharides made up of three identical monosaccharides in variable molar proportions. The antioxidant activity assays showed the carboxymethyl modification could effectively enhance the radical scavenging activity, reducing power, ferrous ion chelating ability and lipid peroxidation inhibition of the native polysaccharide. CMCIP3a demonstrated a more pronounced in vitro anticancer activity on five cancer cells than CIP3a. Moreover, the anticoagulant activity of the carboxymethylated compounds was significantly enhanced. Our findings suggested that carboxymethylation modifications were efficient approaches to enhance the antioxidant, antitumor and anticoagulant activities of natural polysaccharides, since CMCIP3a had more noticeable effects than CIP3a at the same dosage. Therefore, the overall study showed that the structures of the polysaccharides were the most important factors affecting their biological activities, and the structure modification could be an effective tool in the discovery of new bioactive polysaccharides.

## Figures and Tables

**Table 1:** Chemical composition of CIP and its derivative ^#^.

Physicochemical Properties	CIP3a	CMCIP3a
Yield (%)	-	39.15
Carbohydrate (%)	83.23	88.96
Protein (%)	1.75	1.06
Uronic acid (%)	7.22	11.53
Mw (Da)	1.456 × 10^3^	4.023 × 10^3^
Monosaccharide composition (%)		
Arabinose	18.61	15.27
Mannose	17.31	11.05
Glucose	61.59	70.37
Glucuronic acid	0.45	0.56
Galacturonic acid	2.03	2.75

^#^ Values are expressed as mean and SD of three replicates.

## Data Availability

Data is contained within the article.

## Supplementary Materials

The following supporting information can be downloaded at: https://www.mdpi.com/article/10.3390/antiox12010105/s1, Figure S1. NMR spectrum of CIP3a and CMCIP3a, Figure S2. XRD analysis of CIP3a and CMCIP3a.
