# Supplementary material for: Effects of Carboxymethyl Modification on the Acidic Polysaccharides from Calocybe indica: Physicochemical Properties, Antioxidant, Antitumor and Anticoagulant Activities
Source: Antioxidants (Basel). 2022 Dec 31;12(1):105. doi: 10.3390/antiox12010105 (PMC9854956; doi:10.3390/antiox12010105)
Supplement: Supplementary file 1 [file antioxidants-12-00105-s001.zip › antioxidants-2100043-supplementary.pdf]

A

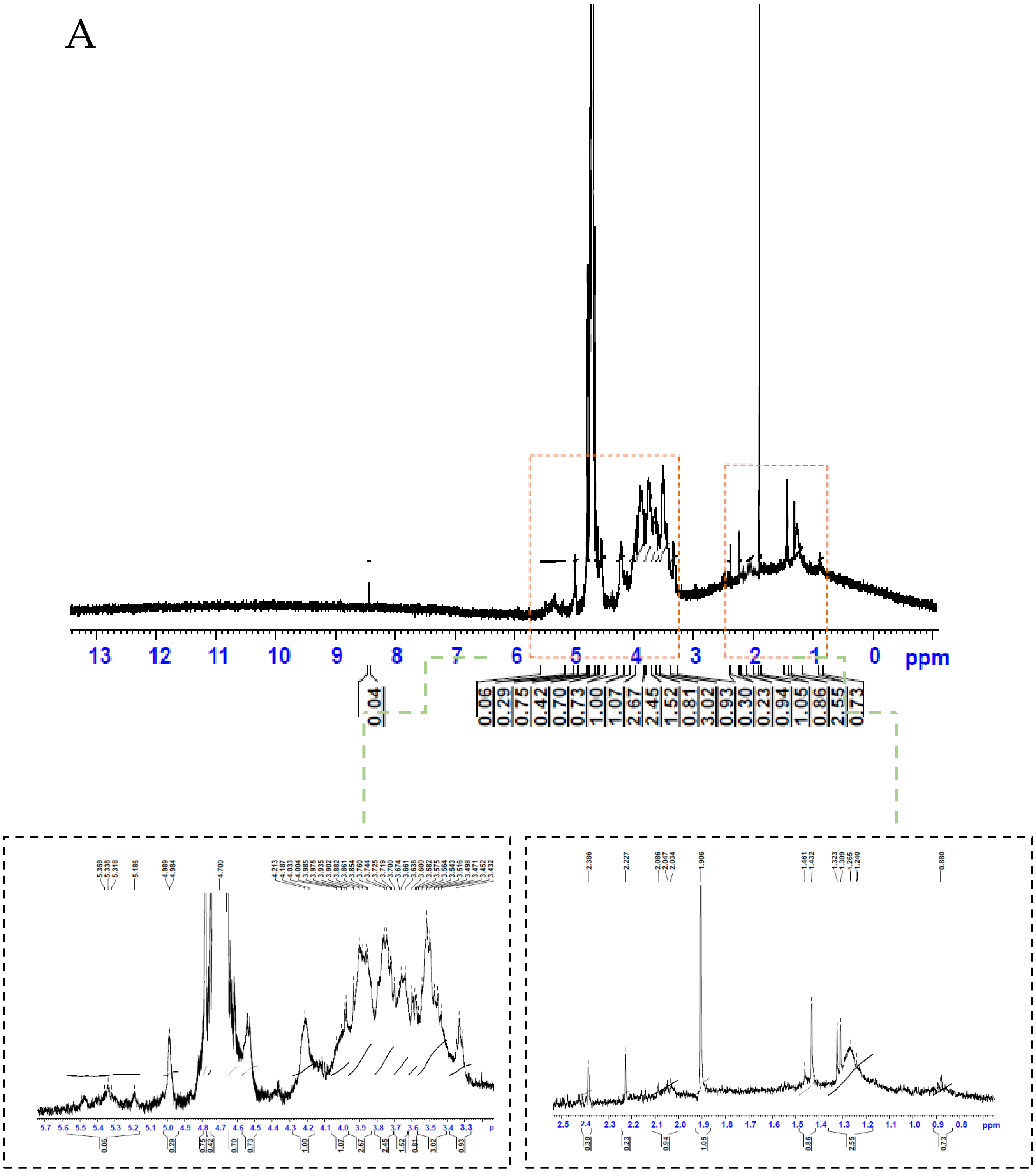

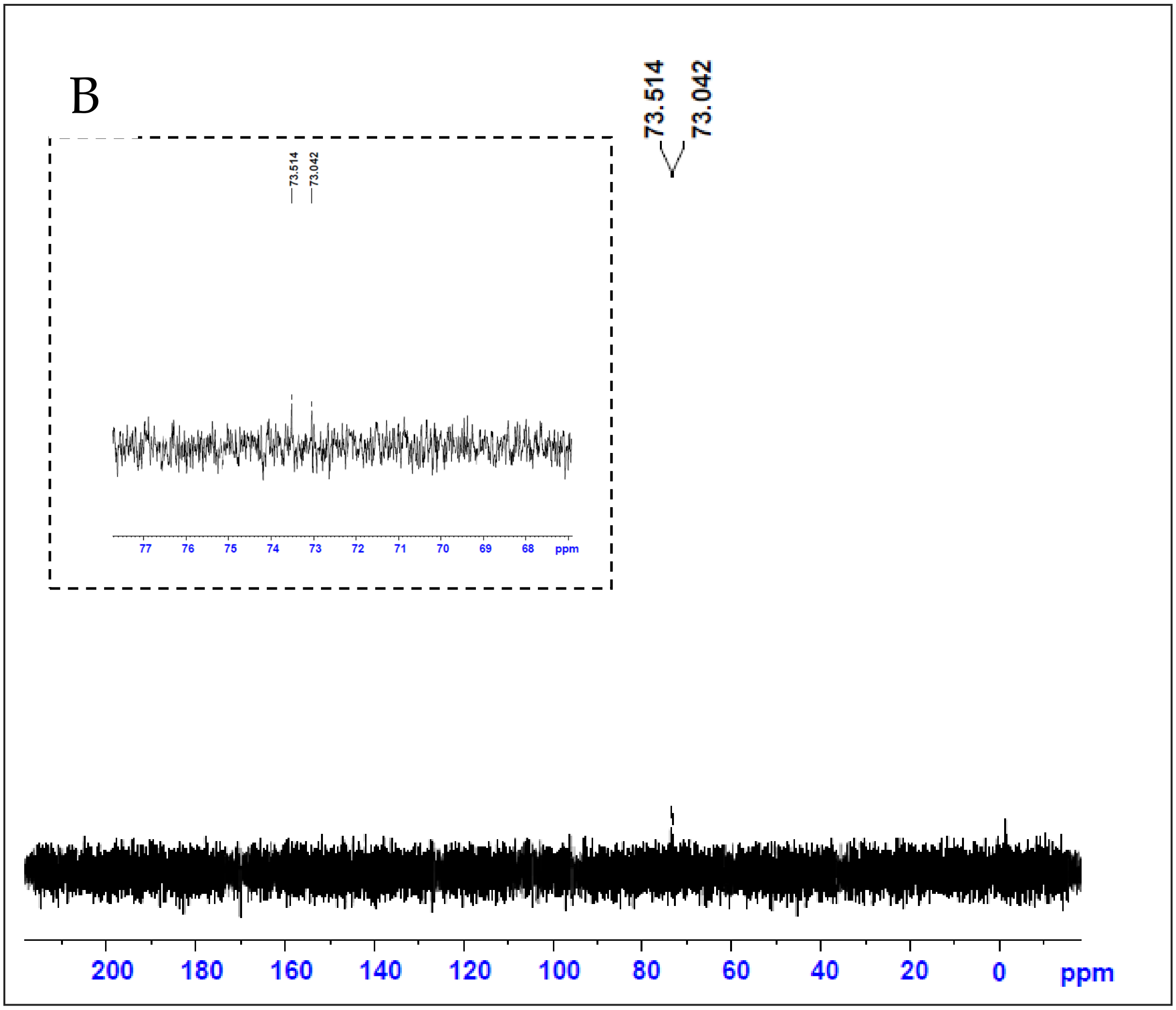
B
73.514
73.042
77 76 75 74 73 72 71 70 69 68 ppm
200 180 160 140 120 100 80 60 40 20 0 ppm

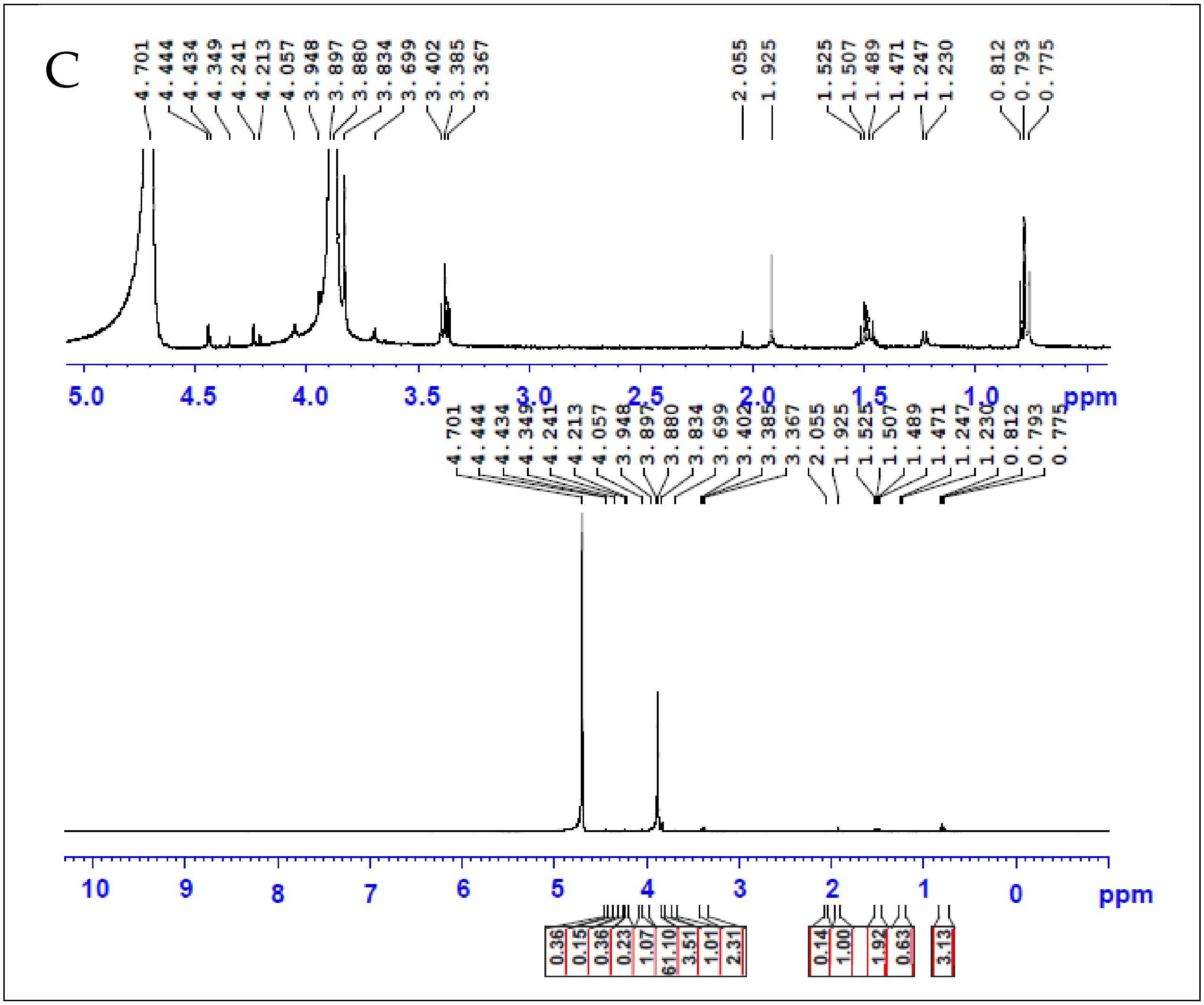
C
4.701
4.444
4.434
4.349
4.241
4.213
4.057
3.948
3.897
3.880
3.834
3.699
3.402
3.385
3.367
2.055
1.925
1.525
1.507
1.489
1.471
1.247
1.230
0.812
0.793
0.775
ppm
0.36
0.15
0.36
0.23
1.07
61.10
3.51
1.01
2.31
0.14
1.00
1.92
0.63
3.13

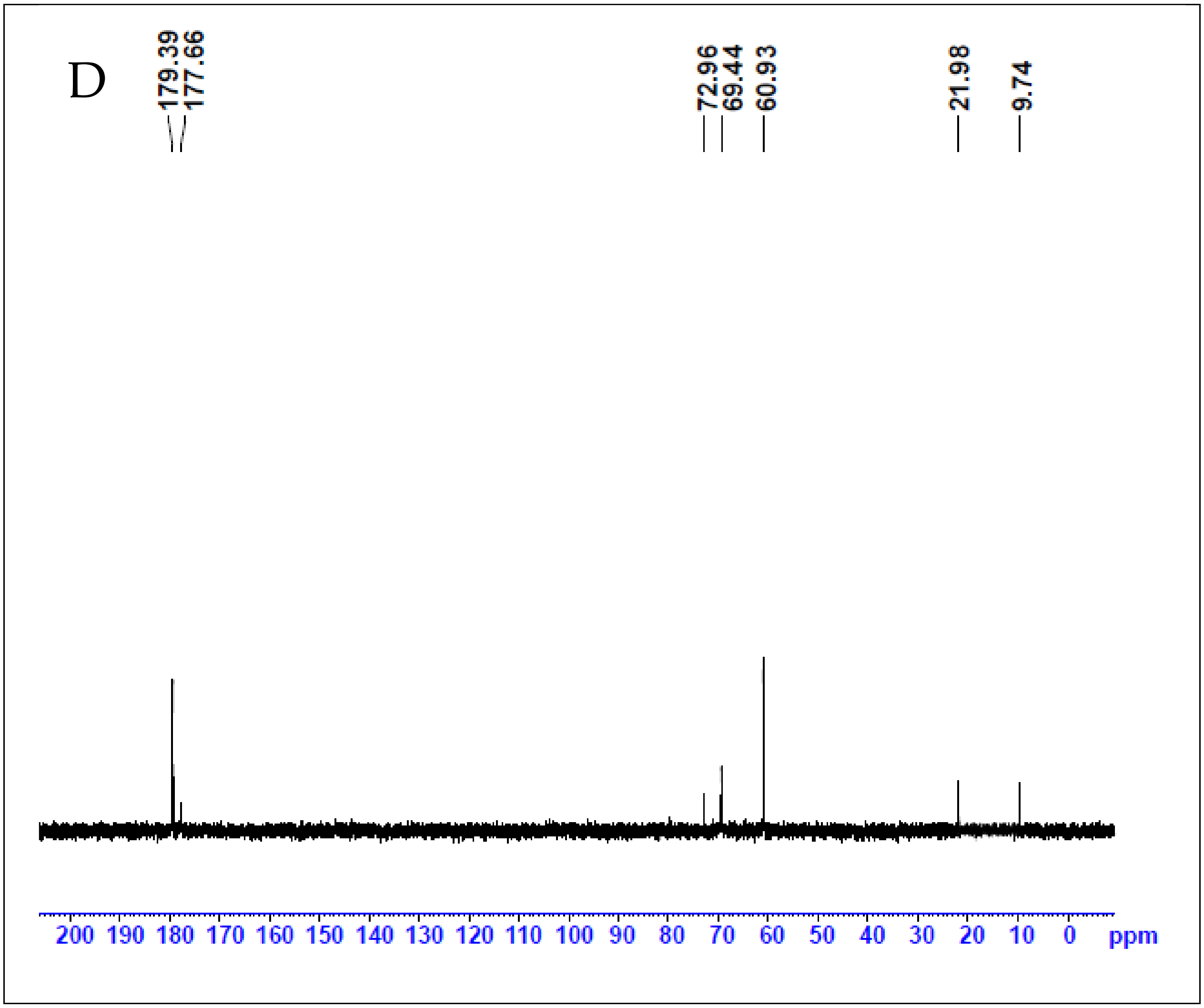

Figure S1. NMR spectrum of CIP3a and CMCIP3a, (A) ^{1}H NMR of CIP3a, (B) ^{13}C NMR of CIP3a, (C) ^{1}H NMR of CMCIP3a, and (D) ^{13}CNMR of CMCIP3a.

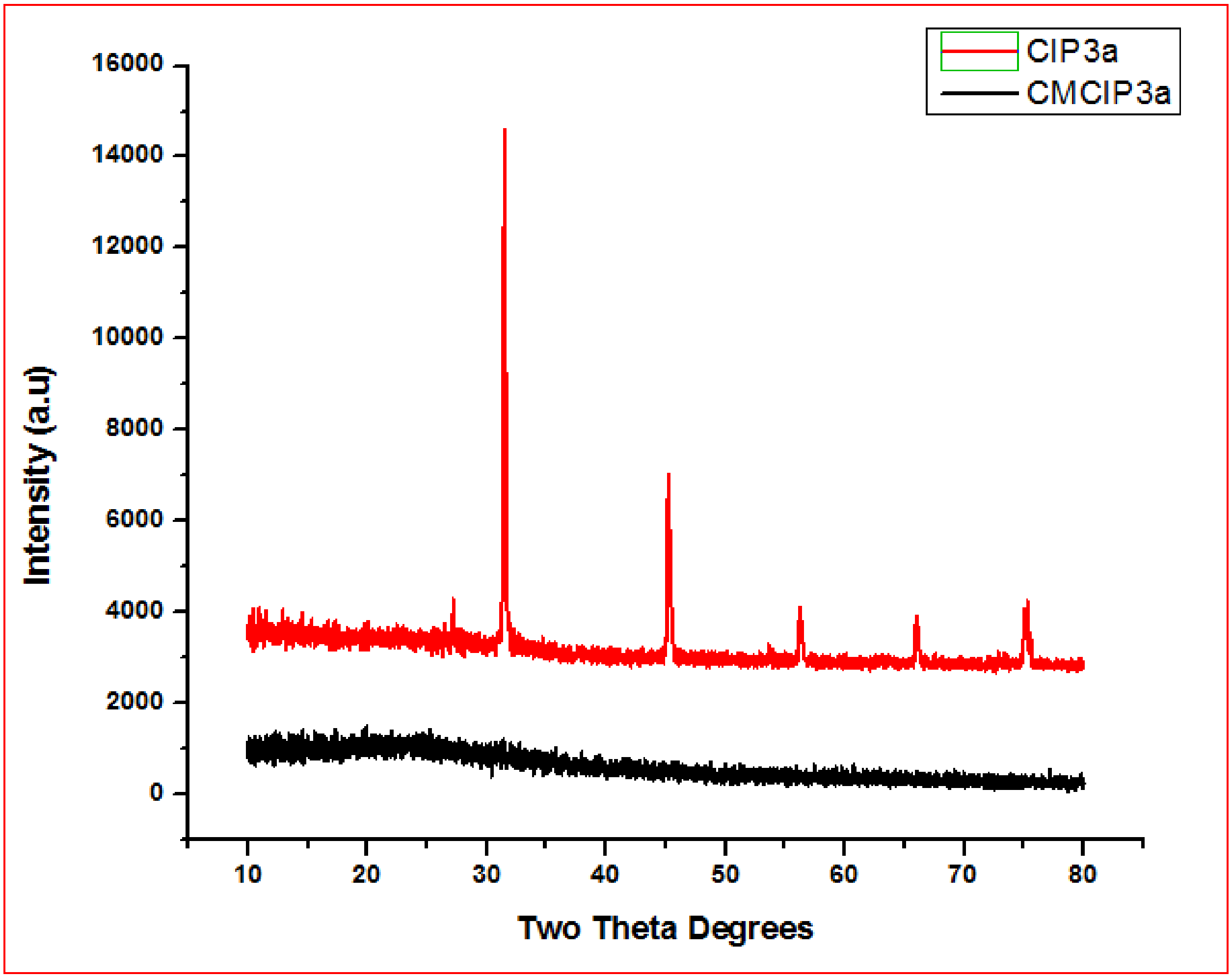

Figure S2. XRD analysis of CIP3a and CMCIP3a.
